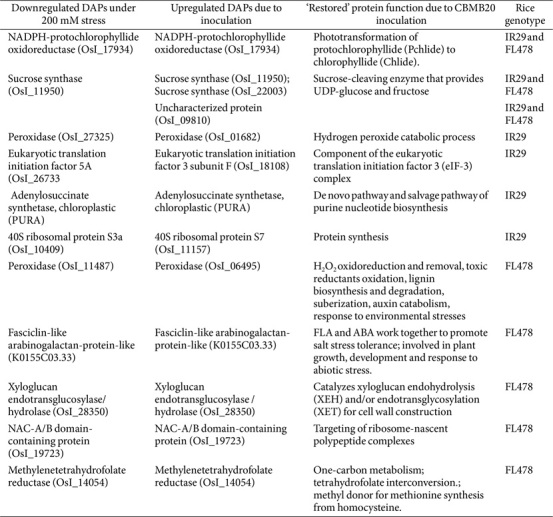# Proteomic Re-Structuring in the Salt-Sensitive Rice Genotype Comparable to Its Salt-Tolerant Counterpart Mediated by an ACC Deaminase-Producing Endophytic Bacteria under Salt Stress

**DOI:** 10.4014/jmb.2412.12074

**Published:** 2025-06-12

**Authors:** Denver I. Walitang, Kiyoon Kim, Yi Lee, Aritra Roy Choudhury, Tongmin Sa

**Affiliations:** 1 Department of Environmental and Biological Chemistry, Chungbuk National University, Cheongju 28644, Republic of Korea; 2 DNA Barcoding Laboratory and College of Arts and Sciences, Romblon State University, Romblon 5505, Philippines; 3 National Forest Seed Variety Center, Korea Forest Service, Chungju 27495, Republic of Korea; 4 Department of Industrial Plant Science and Technology, Chungbuk National University, Cheongju 28644, Republic of Korea; 5 Montana Seed Potato Certification Program, Montana State University, Bozeman, MT 59717, USA; 6 The Korean Academy of Science and Technology, Seongnam, Republic of Korea

**Keywords:** LC-MS/MS, *Methylobacterium oryzae* CBMB20, plant growth-promotion, proteomics, rice, salt stress, salt tolerance

## Abstract

Salt stress creates a combinatorial plant stress encompassing ion toxicity, physiological drought, nutritional imbalance, and oxidative stress. Salinity impacts salt-sensitive and tolerant rice genotypes. Plants also recruit microbes leading to a complex array of microbe-mediated plant responses resulting in a cumulative overall tolerance enhancement to salinity. In this study, label-free proteomics quantification was conducted to assess the responses of rice under salt stress together with microbe-mediated responsive proteomes toward salt stress tolerance. Under salt stress, rice proteomes are mainly influenced by salt stress, rice genotype, and *Methylobacterium oryzae* CBMB20 inoculation. There are common and genotype-specific upregulated and downregulated differentially abundant proteins (DAPs) in the salt-sensitive IR29 and the salt-tolerant FL478 due to salt stress. However, the 1-aminocyclopropane-1-carboxylate (ACC) deaminase-producing *M. oryzae* CBMB20, which regulates ethylene biosynthesis, mediated changes in the salt-stressed IR29 resulting in similar proteomes to that of FL478. Our study provides a mechanistic understanding of the interactions of an ACC deaminase-producing *M. oryzae* CBMB20 where a key feature of the microbe-mediated salt stress response is the restoration of the abundance of many downregulated DAPs in rice under salt stress conditions.

## Introduction

Salinity causes stress in plants via salt-induced water stress affecting water potential and disproportionate salt accumulation in cell compartments causing direct salt toxicity and interference in the absorption of vital nutrients [1-3]. In addition, increasing salt-induced stress leads to the formation of reactive oxygen species (ROS) causing oxidative stress [4]. Salt-induced stresses cause physical and physiological effects from cells to the whole plant triggering stress responses to adapt, acclimate and survive from transient and long-term exposure to salinity [5, 6]. These salt stress responses can be observed in sensitive and tolerant plants, but are more efficient and developed in tolerant varieties [7-9].

Rice (*Oryza sativa*), a critically important carbohydrate source, is generally salt-sensitive with a relatively low threshold [10]. Rice has varying sensitivity to salt stress depending on its growth stage where the seedling stage is very sensitive [11]. In addition, rice presents substantial genetic variabilities within the cultivated gene pool towards salinity tolerance [12, 13]. These genetic differences translate into variable physical and physiological reactions to salt stress such as those seen in the two indica rice genotypes FL478, derived from salt-tolerant populations of recombinant inbred line, and IR29 [14]. FL478 traces its lineage to an original cross between the salt-sensitive, high-yielding maternal parent, IR29, and the salt-tolerant paternal parent Pokkali [14, 15].

In addition, rice forms associations with microorganisms, particularly plant growth-promoting bacteria (PGPB), enhancing growth in non-stressed conditions and even tolerance under stress. PGPB endophytes are of special interest since they inhabit internally and interact directly with the cells of the host plants [16]. As PGPB develop mutualistic interactions with rice, they help improve growth, suppress plant pathogens, and even enhance stress tolerance under stress conditions [17, 18]. For instance, the endophytic, ACC deaminase-producing *Methylobacterium oryzae* CBMB20 is a multifunctional PGPB facilitating plant development under non-stressed and stressed conditions observed in multiple plant species mainly via stress ethylene modulation [19-24]. Rice plants whether sensitive or tolerant to salt, will activate their stress adaptive mechanisms coupled with the recruitment of PGPB leading to the development of microbe-assisted salt tolerance. Many of these plant and plant-bacteria-mediated responses are facilitated by transcripts and proteins, differentially expressed by the plant. At the molecular level, transcriptional and proteomic responses mainly involve alteration of gene expression and translation of proteins [25]. Proteomic changes mediate different physiological responses in varying conditions. Different proteins and protein products such as metabolites resulting from plant-microbe interactions are essential in enhancing stress tolerance [26]. Oftentimes, shifts in the transcriptional level do not exactly correlate to the shifts at the level of proteomes [8]. Also, in a previous connected study [21], inoculation of IR29 and FL478 with *M. oryzae* CBMB20 under normal growing conditions showed inherent similarities and differences in proteomic responses existing between the salt-sensitive and salt-tolerant rice cultivars due to the plant growth-promoting endophytic bacteria *M. oryzae* CBMB20 under non-stress conditions. Therefore, understanding dynamic proteomic changes and protein identification of DAPs associated with salt stress or microbe-mediated stress responses distinguishingly observed in IR29 and FL478 offer a supportive outlook on the molecular mechanisms essential for understanding stress tolerance through plant-microbe interactions.

Thus, the present investigation delves into proteomes and DAPs of two contrasting rice genotypes, the salt-sensitive IR29 and the moderately tolerant FL478 responding to salt stress and those mediated by *M. oryzae* CBMB20 under salt stress conditions. These facilitate the identification of stress-responsive and microbe-mediated responsive proteins providing vital information on proteome-based salt stress tolerance inherently found between the two genotypes and those microbe-mediated responses leading to an overall proteomic response to salinity.

## Materials and Methods

### Bioinoculant Preparation

A plant growth-promoting *M. oryzae* CBMB20 was utilized as the bioinoculant. It was maintained and grown on an ammonium mineral salt (AMS) medium using a carbon source sodium succinate at 0.5%. Bacterial suspension for bioinoculation was prepared by harvesting *M. oryzae* CBMB20 using 0.03 M MgSO_4_ with an OD_600_ ~ 0.8.

### Bacterial Inoculation, Plant Genotypes and Their Growing Conditions

Two contrasting rice genotypes originally developed in the International Rice Research Institute (IRRI), Philippines were used in the study: *Oryza sativa* L. subsp. *indica* cv. IR29 and cv. FL478. Pure seeds of IR29 and FL478 were provided by the Rural Development Administration (RDA), South Korea. The sterilization of seeds was done according to Chatterjee *et al*. (2019) and then germination was initiated by imbibing the seeds with sterile water. Inoculation of bacteria, transplantation of seedlings, and imposition of salt stress were conducted based on Roy Choudhury *et al*. [22]. After initiation of germination, seedling growth was facilitated by transferring the germinated seed into nursery soil and the seedlings were placed under greenhouse conditions using natural illumination. Inoculation with *M. oryzae* CBMB20 was done on 7-day-old rice seedlings. The roots were dipped in bacterial suspension (2 min) and transferred into seedling trays where additional bacterial suspension (5 ml) was added around the root region of the rice seedlings. A sterile 0.03 M MgSO_4_ solution was used in place of the bacterial suspension for non-inoculated plants. The set-up was designed with six treatments: the non-stressed, non-inoculated IR29 (IR_NS_NI), the non-stressed, non-inoculated FL478 (FL_NS_NI), the salt-stressed, non-inoculated IR29 (IR_SS_NI), the salt-stressed, non-inoculated FL478 (FL_SS_NI), the salt-stressed, *M. oryzae* CBMB20 inoculated IR29 (IR_SS_IN), and the salt-stressed, *M. oryzae* CBMB20 inoculated FL478 (FL_SS_IN). Each treatment has three biological replicates.

### Imposition of Salt Stress

After 7 days post bacterial inoculation, 14-day-old seedlings were exposed to salt stress. Thirty seedlings were gathered per replicate and put into mini pots (60 ml capacity) containing 30 ml of 200 mM salt solution. Distilled water was used for the non-stressed treatments. The seedlings were transferred in a growth chamber (DS 54 GLP, SASOL Scientific, Republic of Korea) for 24 h (16-8 h day-night condition, 32°C/28°C day/night temperature, 70% humidity).

### Preparation of Protein Samples Including Extraction, Purification and Digestion

The seedlings (15 days) were harvested and homogenized with liquid nitrogen. For protein extraction, 0.5 g of fresh leaf samples was used following the TCA/Acetone method [27]. The extracted protein was quantified following the Bradford method [28]. Proteins were purified as described by Komatsu *et al*. [29]. Protein samples (100 μg) were alkylated with iodoacetamide and then trypsin enzyme was used for their digestion.

### Protein Analysis with LC-MS/MS

Prepared samples were run through LC-MS/MS analysis using an LTQ Orbitrap mass spectrometer (Thermo Fischer, Germany) coupled with 1100 nano-flow HPLC system (Agilent) composed of an interconnected (three-way tee connector) pre-column, waste line, and the analytical column (C18 AQ, 3 μm, 100 μm × 15 cm, NanoLC, USA). An autosampler loaded 10 μl peptide samples into the C18 trap column (Accaim Pep Map 100, 75 μm × 2 cm, nano Viper C18, 3 μm, Thermo Fischer Scientific, USA). Two mobile phases (A and B) containing 0 and 100% acetonitrile each supplemented with 0.1% formic acid were used. A flow rate of 10 μl/min (10 min; buffer A) followed by a flow elution rate of 300 nl/min (150 min; buffer B) on a 0-50% linear gradient for the trap column desalted and concentrated the peptide samples. The column was washed with buffer B (10 column volumes) every time after elution and then re-equilibrated with buffer A to avoid sample carryover. The data-dependent mode was used for the fragmentation of the five most abundant peaks from the full MS scan with 35% normalized collision energy. The peptide samples were then entered into the mass spectrometer using an electrospray voltage of 2.2 kV. The duration for the dynamic exclusion was 180 sec with 0.5 Da for exclusion mass. The MS spectra have a range of 150-2,000 m/z.

### Peptide and Protein Quantification and Identification

The MS/MS spectra of the detected peptides were cross-referenced against the universal Protein Resource Database (http://www.uniprot.org) with Mascot Daemon (Version 2.4.1, Matrix Science, UK). To identify the peptides, the criteria included were based on monoisotopic mass selection, precursor mass tolerance of ±1.5 Da, fragment mass tolerance of ±0.8 Da, two missed trypsin cleavage, and fixed modification of carbamidomethyl cysteine. The threshold score/expectation value was based on the Mascot ion score threshold (0.5) for accepting individual spectrum calculated for each database search. Identification certainty, as indicated by the false discovery rates (FDR) was calculated by Peptide Validator at 1.0%.

The emPAI method was used for label-free quantitation analysis [30]. The protein identification list was filtered for common contaminant and reverse decoy matches. At least two unique peptides per protein should be present for the protein identification. Proteins observed in at least two technical replicates of three biological trials in each group were used for relative quantification. The average label-free quantification intensity within each biological group was determined using an arithmetic mean statistically evaluated through a two-sided *t*-test. A false discovery rate-based multiple hypothesis testing using Perseus statistics software was used to correct the calculated *p*-value (<0.05). Linear regression analysis was used for data normalization.

### Bioinformatics Analysis

Fold changes (FC) of the DAPs were analyzed through treatment comparisons using abundances. Significance was detected through *t*-test with *p*-value <0.05. Differentially abundant proteins are significantly higher if the FC is greater than 1.5 and significantly lower when the FC is less than 0.6. Gene ontology (GO) terms of the significantly abundant and less abundant DAPs were categorized based on the biological process they are involved in, cellular components where they are located, and their molecular functions using DAVID Bioinformatics resources 6.8 (https://david.ncifcrf.gov). We used default settings in DAVID, which include a background of the whole genome of *Oryza sativa* (the reference organism used for protein identification) and the default significance threshold of EASE score (a modified Fisher’s Exact *p*-value) ≤ 0.1 for enrichment. For KEGG pathway mapping, functional annotations were performed using the DAVID-integrated KEGG tool, which assigns proteins to pathway based on their KEGG Orthology (KO) terms also determined through the KEGG database (http://www.genome.jp/kegg/pathway.html).

### Visualization and Statistical Analysis

The research experimental set-up is in a completely randomized block. The web-based tool *clustvis* [31] was used to generate heatmaps and their related dendrogram. Protein abundance data were also log2-transformed and row-wise centered and scaled (*i.e.*, standardization to zero mean and unit variance for each protein) to emphasize relative abundance patterns across treatments and genotypes. Hierarchical clustering was performed using Euclidean distance and average linkage. Principal component analysis (PCA) of the overall proteins was done in R using Rforproteomics packages. Prior to PCA, protein abundance values were log2-transformed to reduce heteroscedasticity, and then mean-centered and scaled to unit variance to ensure equal contribution of variables to the principal components. The web-based tool Venny [32] was used to generate the Venn diagrams.

## Results

### Overall Proteomes of *Oryza sativa* spp. *indica* cv. IR29 and cv. FL478 under Non-Stress and Salt Stress Conditions and with and without *Methylobacterium oryzae* CBMB20 Inoculation

**Fig. 1:**
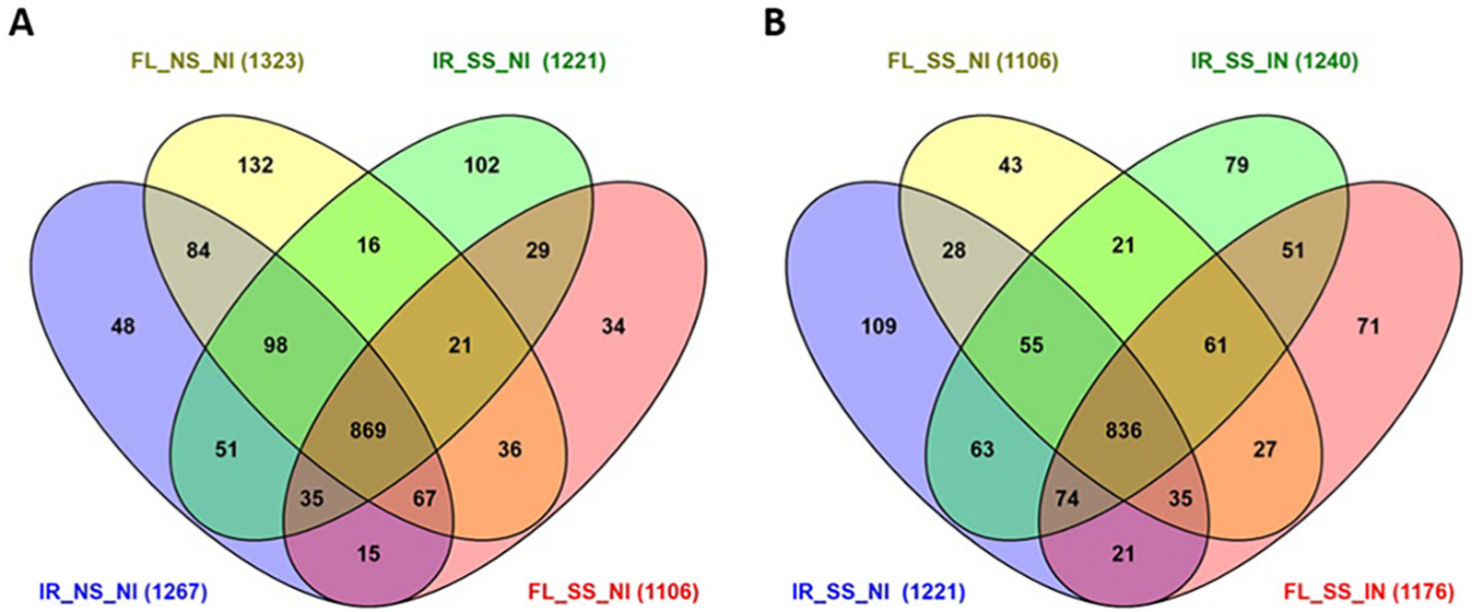
Venn diagrams of total, common and unique proteins in IR29 and FL478 under (A) non-inoculation and salt stressed and non-stressed conditions, and (B) *Methylobacterium oryzae* CBMB20 inoculation and non-inoculation under stress conditions.

**Fig. 2:**
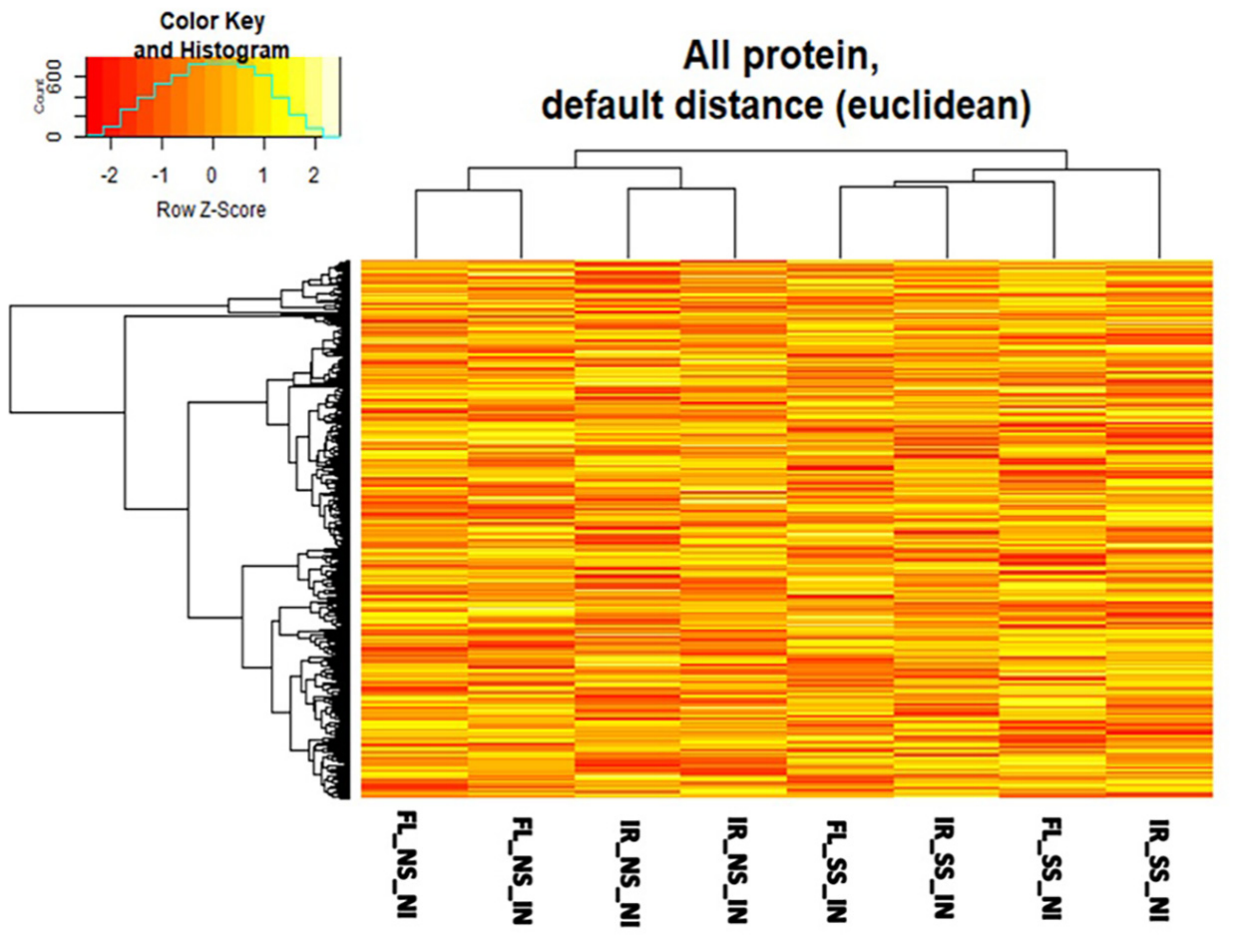
Heatmap comparison of overall proteomes in the different treatments.

**Fig. 3:**
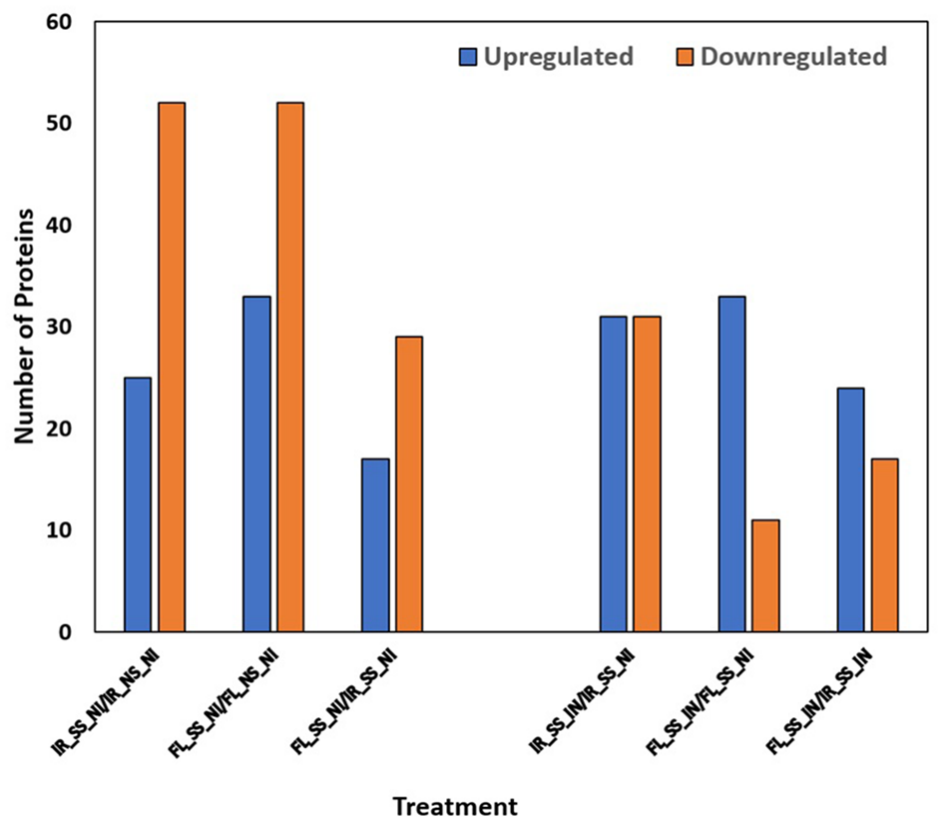
Summary of significant DAPs between treatment comparisons.

Through LC-MS/MS quantitative proteomics, overall proteomes detected across all treatments included a total of 1267, 1323, 1221, 1106, 1240, and 1176 were detected in IR-NS-NI, FL-NS-NI, IR-SS-NI, FL-SS-NI, IR-SS-IN, and FL-SS-IN, respectively (Fig. 1). The number of common proteins found in treatment comparisons concerning salt stress (Fig. 1A) ranged from 65.68% - 78.57% while those treatment comparisons affected between *M. oryzae* CBMB20 inoculated and salt-stressed plants ranged from 67.42% - 75.59% (Fig. 1B), relative to their individual total proteins. Interestingly, comparison in terms of dendrogram and heatmap analyses of all proteomes found in each treatment showed that, in general, salt stress is the main factor affecting proteome composition and abundance across all treatments where non-stress (NS) treatments separated from salt-stressed (SS) treatments. Furthermore, under non-stress conditions, genotype followed by inoculation were the interacting factors affecting proteomes of the treatments. However, the increased importance of inoculation became prominent when rice plants experienced salt stress where inoculation with the multifaceted *M. oryzae* CBMB20 greatly affected the proteomes of rice plants followed by the effect of genotypes (Fig. 2). It is noteworthy to point out that the IR_SS_IN treatment was more similar to the FL_SS_IN in terms of proteomes than the FL_SS_NI treatment. The change in proteome composition under salt stress upon application of *M. oryzae* CBMB20 indicates the impact and potential contribution of the bioinoculum in rice under stress conditions. Principal component analysis (Fig. S1) showed similar trends to that of the heatmap and dendrogram in Fig. 2, with a combined 49.2%overall variability with PC1 explaining 26% and PC2 showing 23.2%. A few trends were also observed in the number of significant differentially abundant proteins (DAPs) (Fig. 3). Salt stress has increased the number of downregulated DAPs while *M. oryzae* CBMB20 inoculation showed a balanced or more abundant upregulated DAPs under salt stress conditions. In detail, there were 26 and 53 significantly upregulated and downregulated DAPs, respectively, in IR-SS-NI vs IR-NS-NI treatment comparison. Similarly, there were 34 and 53 significantly upregulated and downregulated DAPs, respectively, in FL-SS-NI vs FL-NS-NI comparison. Significantly upregulated and downregulated DAPs in IR-SS-IN vs IR-SS-NI include 32 and 32 proteins, respectively while 34 and 12 proteins were observed to be significantly upregulated and downregulated, respectively, in FL-SS-IN vs FL-SS-NI treatment comparison. The lists of upregulated and downregulated DAPs between treatment comparisons are shown in Table S1.

### Gene Ontology (GO) Terms of DAPs between Treatment Comparisons under Salt Stress

**Fig. 4:**
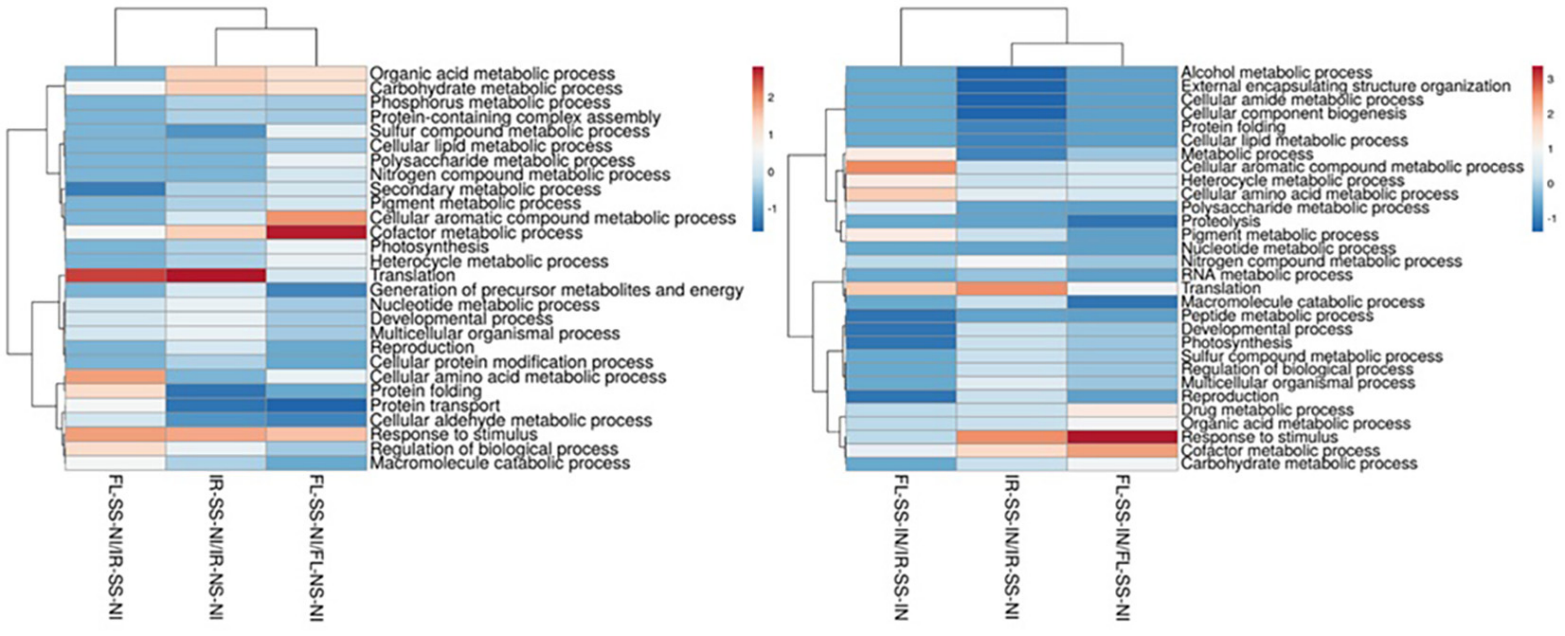
Gene ontology terms of DAPs in salt-stressed versus non-stressed rice (left) and salt-stressed, inoculated rice vs salt-stressed, non-inoculated rice (right) based on biological process.

**Fig. 5:**
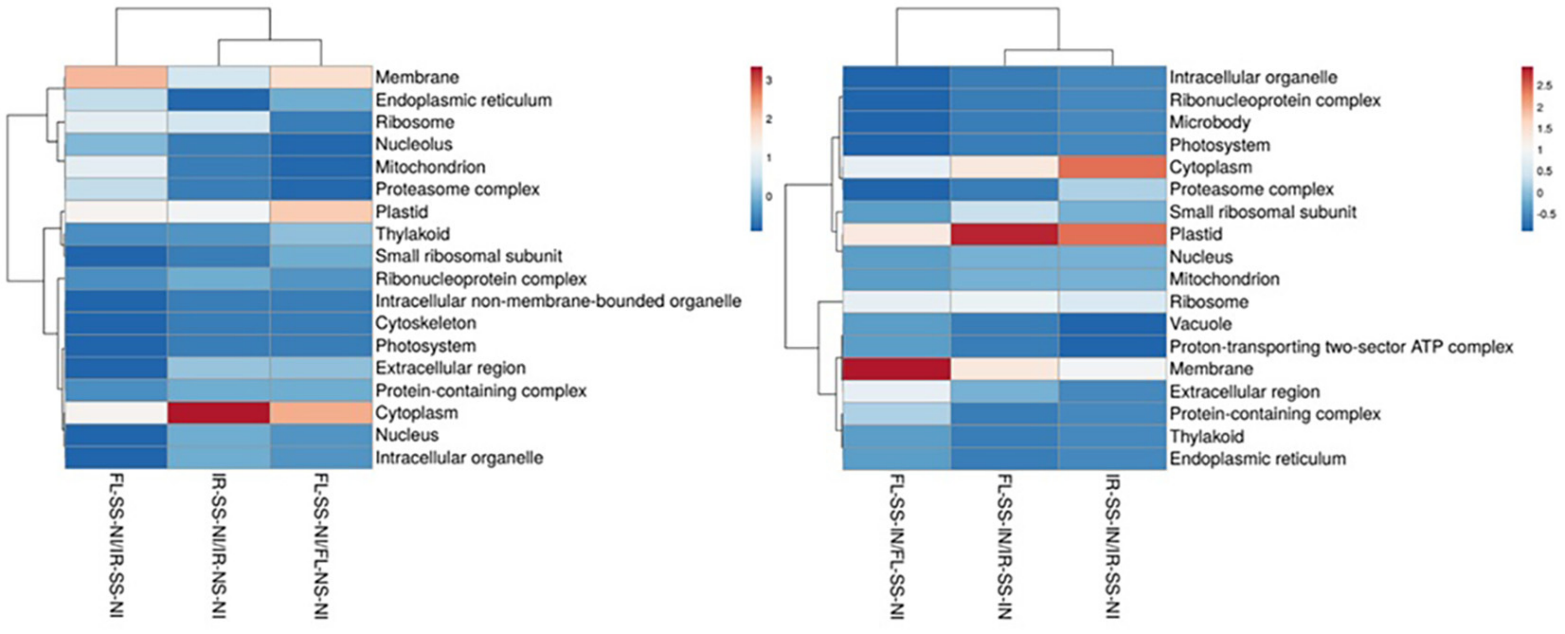
Gene ontology terms of DAPs in salt-stressed versus non-stressed rice (left) and salt-stressed, inoculated rice vs salt-stressed, non-inoculated rice (right) based on cellular component.

**Fig. 6:**
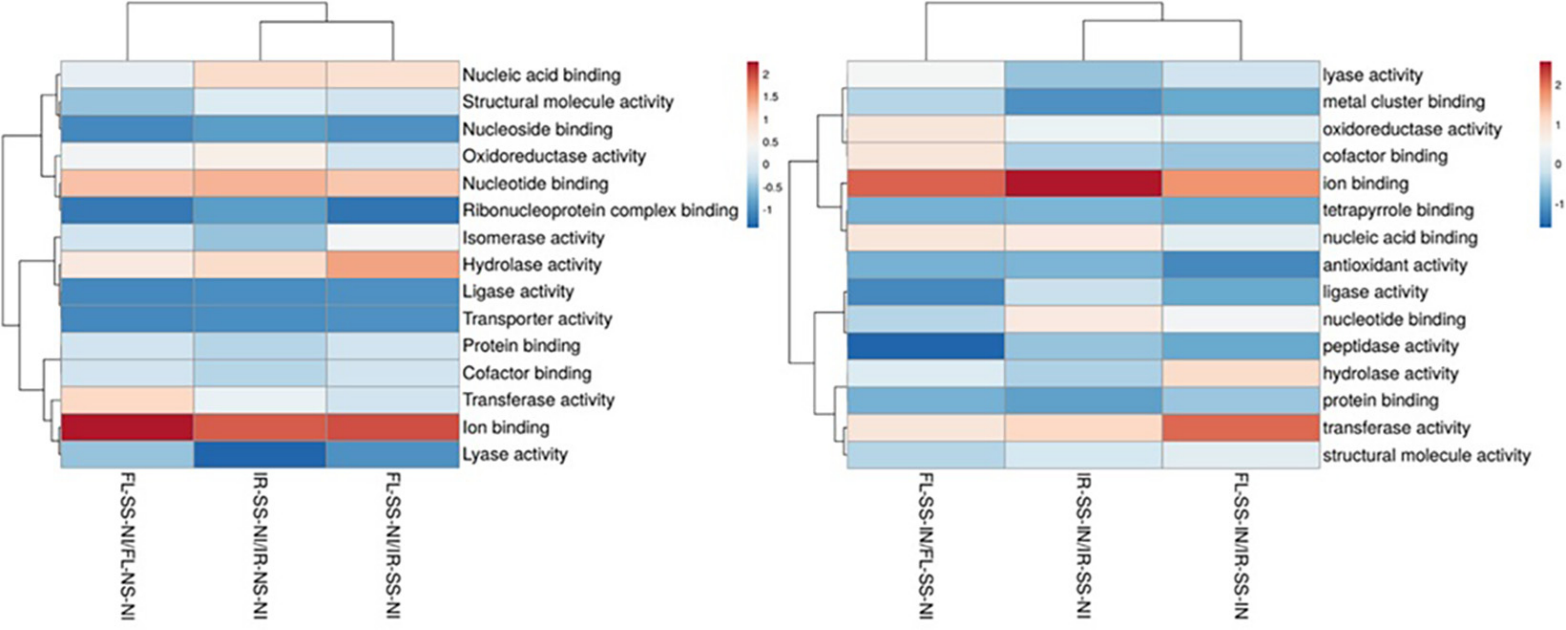
Gene ontology terms of DAPs in salt-stressed versus non-stressed rice (left) and salt-stressed, inoculated rice vs salt-stressed, non-inoculated rice (right) based on molecular process.

The treatment comparisons between IR-SS-NI vs IR-NS-NI, FL-SS-NI vs FL-NS-NI, and FL-SS-NI vs IR-SS-NI could infer intrinsic genotypic and phenotypic variabilities existing between IR29 and FL478 as shown in their expressed proteomes associated with mechanisms of salt tolerance and physiological responses when these contrasting rice genotypes are grown in salinized media. The more abundant and the less abundant significant DAPS were processed for GO cluster analysis determining the biological process involved in (Fig. 4), their location in the cell or the cellular structure they are attributed to (Fig. 5), and their molecular functions (Fig. 6) with details of gene ontology of different DAPS in different treatment comparisons (Tables S2, S3, and S4). Under 200 mM salt stress conditions, there are potential differences between the salt-stressed IR29 and FL478. More abundant DAPs in IR29 under salt stress cluster in translation (8.33%), response to stimulus (6.25%), cofactor metabolic process (5.56%), carbohydrate metabolic process (5.56%), and organic acid metabolic process (5.56%). The majority of DAPs in FL478 under salt stress include the cofactor metabolic process (7.38%), cellular aromatic compound metabolic process (6.04%), response to stimulus (5.37%), carbohydrate metabolic process (4.70%), and organic acid metabolic process (4.70%). The main difference is the dominance of translation-related proteins in the salt-stressed IR29 and the abundance of cellular aromatic compound metabolic process-related proteins in the salt-stressed FL478 (Fig. 4A). The majority of the significant DAPs in the salt-stressed IR29 are mainly localized in the cytoplasm (25.37%), followed by the plastids (11.94%), membranes (8.96%) and ribosomes (8.95%). The localization of the DAPs for the salt-stressed FL478 is equally distributed in three major regions, the cytoplasm (19.70%), the plastids (18.18%), and the membranes (16.67%) (Fig. 5A). The DAPs for both the salt-stressed IR29 and FL478 are attributed to major molecular functions including hydrolase activity, ion binding, nucleic acid and nucleotide binding, oxidoreductase and transferase activity (Fig. 6A).

### Gene Ontology (GO) Terms of DAPs between Treatment Comparisons under Salt Stress and Inoculation with *M. oryzae* CBMB20

Candidate DAPs in IR29 and FL478 necessitating microbe-mediated responses under severe salt stress conditions could be inferred through treatment comparisons of IR-SS-IN vs IR-SS-NI, FL-SS-IN vs FL-SS-NI, and FL-SS-IN vs IR-SS-IN. The GO terms of the significant DAPs of the inoculated IR29 and FL478 plants under 200 mM salt stress were clustered in terms of biological process (Fig. 4), cellular component (Fig. 5), and molecular function (Fig. 6) with the details of gene ontology of different DAPS in different treatment comparisons (Tables S5, S6, and S7).

Microbe-mediated proteome responses of salt-stressed IR29 and FL478 seem to mainly differ in terms of proportions and locations as observed in the GO terms related to cellular components, molecular functions, and biological processes. For biological function, the DAPs in the salt-sensitive IR29 are more evenly distributed in terms of proportion where response to stimulus (7.75%) and translation (7.75%) related proteins are more abundant followed by cofactor metabolic process (6.20%) and nitrogen compound metabolic process (4.65%). In contrast, the biological functions of DAPs in FL478 have unequal proportions where the majority of the DAPs are related to response to stimulus (11.69%), cofactor metabolic process (9.09%), organic acid metabolic process (5.19%), carbohydrate metabolic process (5.19) and translation (5.19%). The majority of the significant DAPs in the salt-stressed IR29 are mainly localized in the plastid (20.45%), cytoplasm (20.45%), membranes (11.36%), and ribosomes (9.09%). The localization of DAPs for the salt-stressed FL478 is mainly concentrated in the membranes (24.13%), plastids (13.79%), cytoplasm (10.34%), ribosomes (10.34%), and the extracellular region (10.34%). Molecular functions of the significant DAPs in IR29 are mainly related to ion binding (19.81%), transferase activity (12.26%), nucleic acid binding (10.38%) and nucleotide binding (10.38%). For FL478, there is an equal distribution of DAPs related to ion binding (14.29%), transferase activity (9.52%), nucleic acid binding (9.52%), oxidoreductase activity (9.52%, and cofactor binding (9.52%).

### The Influence of Salt Stress on IR29 and FL478 DAPs, Their Similar and Genotype-Specific Proteomic Responses

Severe salt stress may profoundly affect IR29 and FL478 and this could be detected on their global proteomics. As observed in Fig. 3, there are more downregulated proteins in both IR29 and FL478 as a result of salt stress. Additionally, Table 1 (Tables S8 and S9) shows common upregulated and downregulated proteins indicating similar reactions of IR29 and FL478 under 200 mM salt stress imposition. There are five common upregulated proteins in IR29 and FL478 responding to severe salt stress. Three common DAPs are associated with stress and defense including the Usp domain-containing protein (OsI_02150), glutaredoxin domain-containing protein (OsI_37657), and aldehyde dehydrogenase (NAD^+^) (OsI_31526), The peptidyl-prolyl cis-trans isomerase (OsI_00098) is associated with protein folding but is responsive to salt stress. An uncharacterized protein (A2X9N1) was also detected in both IR29 and FL478. The universal stress protein (Usp) domain-containing protein (OsI_02150) has a fold change (FC) upregulation of 3.93 in IR29 and 4.82 in FL478. The glutaredoxin domain-containing protein (OsI_37657) (IR29-2.11, FL478-2.11), aldehyde dehydrogenase (NAD^(+)^) (OsI_31526)(IR29-2.22, FL478-3.28) and peptidyl-prolyl cis-trans isomerase (OsI_00098) (IR29-1.91, FL478-2.54) all have higher FC values. The uncharacterized protein (A2X9N1) is greatly upregulated in both IR29 (FC-4.85) and FL478 (7.45). On the other hand, diverse cellular functions are downregulated through proteins in both IR29 and FL478 including photosynthesis - NADPH-protochlorophyllide oxidoreductase (OsI_17934), carbohydrate metabolism - sucrose synthase (OsI_11950), transcription and protein synthesis related - Histone H4 (OsI_04340), CCT-eta (OsI_24243), organelle organization - Clu domain-containing protein (OsI_06619), cell structure-related -UDP-glucose 6-dehydrogenase (OsI_13603), and metabolism - adenylosuccinate synthetase, chloroplastic (PURA).

### Proteomics-Based Mechanisms of Microbe-Mediated Salt Stress Tolerance and Restored Protein Functions Conferred by *M. oryzae* CBMB20 in Rice

The bioinoculation of *M. oryzae* CBMB20 in IR29 and FL478 and their subsequent exposure to severe salt stress conditions allows us to investigate potential target proteins essential for microbe-mediated salt stress tolerance in rice (Table 2). We have identified three common proteins with significantly downregulated abundance under severe salt stress where inoculation with *M. oryzae* CBMB20 significantly upregulated their fold change in IR29 and FL478 even under severe salt stress conditions. A protein related to phototransformation - NADPH-protochlorophyllide oxidoreductase (OsI_17934) which was observed to be downregulated under salt stress in both IR29 and FL478 was significantly overexpressed upon inoculation of *M. oryzae* CBMB20. This is the same with an enzyme related to carbohydrate metabolism - sucrose synthase (OsI_11950) with restored function upon *M. oryzae* CBMB20 inoculation. The fold change of the uncharacterized protein - uncharacterized protein (OsI_09810) was also significantly increased in *M. oryzae* CBMB20 inoculated, salt-stressed plants.

In addition, genotype-specific DAPs that were significantly downregulated during salt stress were seen to have significant upregulation in *M. oryzae* CBMB20 inoculated rice plants. There were four proteins with recovered and significantly upregulated abundance in the salt-sensitive IR29 involved in ROS scavenging - peroxidase (OsI_27325), purine biosynthesis - adenylosuccinate synthetase, chloroplastic (PURA), and protein synthesis -eukaryotic translation initiation factor 5A (OsI_26733) and 40S ribosomal protein S3a (OsI_10409). Genotype-specific DAPs observed in FL478 with recovered abundance include an isotype of the peroxidase enzyme observed in IR29 - Peroxidase (OsI_11487), an abiotic stress-responsive protein - Fasciclin-like arabinogalactan-protein-like (K0155C03.33), a protein for cell wall construction - Xyloglucan endotransglucosylase/hydrolase (OsI_28350), protein synthesis-related - NAC-A/B domain-containing protein (OsI_19723), and a protein involved in one-carbon metabolism - methylenetetrahydrofolate reductase (OsI_14054).

## Discussion

The current investigation determines the influence of severe salt-induced stress and the response of the sensitive (IR29) and moderately tolerant (FL478) rice and how bioinoculation using a multifaceted PGP endophytic bacterium, *M. oryzae* CBMB20, facilitates microbe-mediated salt stress tolerance. Global proteomic differences between the treatments were compared. To establish the impact of salt stress on IR29 and FL478, the non-stressed, non-inoculated rice was compared to the salt-stressed, non-inoculated rice (IR29_SS_NI vs IR29_NS_NI; FL478_SS_NI vs FL478_NS_NI). To assess microbe-mediated salt stress tolerance, the salt-stressed, non-inoculated plants were compared to the salt-stressed, *M. oryzae* CBMB20 inoculated plants (IR29_SS_IN vs IR29_SS_NI; FL_SS_IN vs FL_SS_NI). Additional information could also be derived from comparisons of FL_SS_NI vs IR_SS_NI and FL_SS_IN vs IR_SS_IN.

### Severe Salt Stress and *M. oryzae* CBMB20 Inoculation under Severe Salt Stress Change Global Proteomes of IR29 and FL478

The adverse and sometimes detrimental action of salinity in plants is a combinatorial stress of sodium ion (Na^+^) toxicity, salt-induced osmotic and oxidative stresses complicated by an imbalance of ionic homeostasis and deficiency in nutrients [1, 3, 33]. This combinatorial stress condition individually, cumulatively, or even synergistically induces rice to respond causing dynamic shifts in plant proteomics. Adaptations to salt stress differentiate glycophytes and halophytes allowing halophytes to even thrive in severe stress conditions [33-35], however, understanding responses between tolerant and sensitive genotypes at the molecular level such as at the proteome level potentially allows us to extend the tolerance of important crops, which are generally sensitive to salt stress.

The present study shows that global proteomic differences occur in the tolerant and sensitive rice cultivars under non-stressed and stressed conditions and between the inoculated and non-inoculated rice. Looking into the interactions of genotype, salt stress, and bioinoculation on the global proteomes of IR29 and FL478, severe salt stress became a main factor in clustering stressed plants and non-stress plants showing an overwhelming proteomic restructuring of rice plants responding to salt stress. The plant genotype becomes the second most important factor affecting global proteomic differences then inoculation as observed in the non-stressed plants. Furthermore, for the salt-stressed plants, it is notable that the global proteomes of the inoculated IR29 under salt stress is more similar to the inoculated, salt-stressed FL478 compared to the non-inoculated, salt-stressed FL478. This potentially indicates more significant changes in the global proteomes of the inoculated IR29 under stress comparable to the proteomes of the inoculated, salt-stressed FL478. As was previously reported [21], *M. oryzae* CBMB20 bioinoculation on the two rice genotypes under non-stressed conditions has also led to changes in the global proteomes of IR29 and FL478 expanding the proteome’s gene ontology terms observed in both plants with genotype-specific proteomic changes, especially on the significantly regulated DAPs. In the present study, salt stress and inoculation of plants followed by salt stress imposition have also dramatically altered the proteomes of the two rice genotypes. However, the higher similarity of proteomes of inoculated and stressed IR29 and FL478 indicates a more profound microbe-mediated response in IR29. A similar observation was also seen in a previous investigation where bioinoculation with *M. oryzae* CBMB20 led to greater bacteria-dependent changes in IR29 under stress conditions leading to greater improvement of physiological parameters in the stressed IR29 compared to FL478 [36]. Another similar observation between inoculated and stressed IR29 and FL478 showed greater responses of the sensitive rice cultivar to bioinoculation compared to the tolerant genotype leading to greater production of osmolytes in IR29, although the inoculant used was *Brevibacterium linens* RS16, another bacteria that modulate salt stress due to its ACC deaminase production [37]. These observations indicate that inoculation with *M. oryzae* CBMB20 on the IR29 and FL478 under salt stress conditions leads to microbe-mediated global proteomics changes with more effective microbe-mediated responses on the salt-sensitive rice cultivar.

The changes in global proteomes under stress could be traced to the stress-responsive transcriptomics changes. Salt stress induces transcriptional changes in rice cultivars within minutes to hours after imposition of salt stress with potential differences in the gene expression level and even timing responses [38]. In general, a succession of responses can be observed in salt-stressed plants within a time frame of minutes to weeks following initiation of salt-induced stress [1, 39]. The changes in global proteomes between IR29 and FL478 are observed in our study. These differences were also observed in a previous study in terms of transcriptional and translational levels, although the genotypes being compared are IR29 and Pokkali, the original paternal parent of FL478 [8]. Theoretically, FL478 and IR29 share 50% of their genome as IR29 is the original maternal parent of FL478, and the remaining 50% is from the original paternal parent, Pokkali [15, 40]. However, prominent response differences between IR29 and FL478 are evident in a previous study even at the transcriptional level [14]. The bioinoculation with the endophytic PGPB *M. oryzae* CBMB20 before the imposition of salt stress resulted in additional changes and responses in IR29 and FL478. Although transcripts and proteomes do not always correlate due to post-transcriptional regulations and changes, the differences in the proteomes of IR29 and FL478 may also be a result of the loading efficiency of mRNA onto polysomes [8] and inherent differences in the timing and efficiency of responses to salt stress [38], and other post-transcriptional regulation leading to differences in the observed proteomes. The details on the specific DAPs responsive to salt stress and those microbe-mediated responsive DAPs are discussed below.

### GO Terms of DAPs in IR29 and FL478 under Severe Salt Stress and under Bioinoculation with *M. oryzae* CBMB20 Followed by Salt Stress

There are genotype-specific differences and similarities observed when comparing GO terms between IR29 and FL478 in terms of biological processes, cellular compartmentalization and molecular functions. Our results show that under severe salt stress, DAPs related to translation are more abundant in IR29 while those in FL478 are associated with cellular aromatic compound metabolic process and cofactor metabolic process. Other DAPs are also associated with the organic acid metabolic process, response to stimulus, and carbohydrate metabolic process observed in IR29 and FL478. However, if we look into the cellular component in which these DAPs are located, there is an overwhelming abundance of DAPs located in the cytoplasm in IR29 followed by the plastids, membranes and ribosomes in contrast to a more balanced distribution of DAPS in the cytoplasm, plastid and membranes in FL478.

Under salt stress conditions, distinctive differences can be observed between IR29 and the tolerant Pokkali where Pokkali was observed to be more effective in enhancing cell wall integrity, detoxifying ROS, translocating molecules and maintaining photosynthesis [8]. Theoretically, these observations should also be observed in FL478 as Pokkali is the putative donor of salt tolerance in FL478. Generally, salt stress causes an upregulation of responses [41]. However, our observations showed that the sensitive and moderately tolerant genotypes have more downregulated DAPs. This is potentially due to the severity and relatively long duration of severe stress imposition. The current study also shows that FL478 has more significantly upregulated DAPs compared to IR29 indicating its more effective salt stress responses. It is also possible that the presence of more significantly upregulated DAPs in FL478 indicates recovery of downregulated DAPs in the initial response to salinity attaining the same expression or even higher prior to salt stress as observed in its paternal parent, Pokkali [38]. During the early stages of stress, there is an increased protein synthesis and protein turnover which eventually shifts to the induction of stress-responsive transcripts and then the production of proteins with defense-related functions [38]. The overrepresented DAPs related to translation located in the ribosomes observed in IR29 observed in this study probably indicate the delayed response of the salt-sensitive genotype while transitioning to stress-responsive and defense-related functions observed in FL478 with enhanced metabolic-related processes.

The GO terms of DAPs in bioinoculated IR29 and FL478 under salt stress conditions reflects the overall global proteomic trends observed. In general, trends in the gene ontology observed in the inoculated, salt-stressed FL478 are also observed in the inoculated, salt-stressed IR29 with slight differences in abundance. These similar patterns were also observed in the cellular localization, molecular functional roles and biological processes of the gene ontology terms of DAPs.

### Proteome Responses to Severe Salt Stress in IR29 and FL478 Rice Genotypes: Salt Stress-Responsive Proteins

Severe and long-term salt stress adversely affects similar mechanisms in plants, whether they are sensitive or tolerant, and the differences in responses determine their stress tolerance in contrast to clear adaptations of halophytes [34]. Key features in the appearance of salt tolerance are the more efficient and effective management of stress responses rather than the creation of adaptations uniquely seen only in specialized plants such as halophytes [38, 39]. Severe salt stress in our study has shown specific salt stress-responsive proteins common to both IR29 and FL478 indicating similar features and processes influenced by salt stress in terms of proteins. We have detected five DAPs significantly upregulated in both IR29 and FL478. These include the Usp domain-containing protein (OsI_02150), glutaredoxin domain-containing protein (OsI_37657), aldehyde dehydrogenase (NAD^(+)^) (OsI_31526) and peptidyl-prolyl cis-trans isomerase (OsI_00098) as well as an uncharacterized protein (A2X9N1). The universal stress protein (Usp) domain-containing protein (OsI_02150) has a fold change (FC) upregulation of 3.93 in IR29 and 4.82 in FL478 indicating its clear importance in salt stress response in both genotypes. The glutaredoxin domain-containing protein (OsI_37657), aldehyde dehydrogenase (NAD^(+)^)(OsI_31526) and peptidyl-prolyl cis-trans isomerase (OsI_00098) also have relatively higher FC values. It is also noteworthy to point out that the uncharacterized protein A2X9N1 significantly upregulated in FL478 has an FC value of 7.45, the most upregulated protein detected in IR29 and FL478 induced by salt stress. Furthermore, the greater FC values in FL478 compared to the IR29 of these stress-related proteins such as in the case of Usp domain-containing protein, aldehyde dehydrogenase, peptidyl-prolyl cis-trans isomerase and the uncharacterized protein A2X9N1 supports the notion that the moderately salt tolerant FL478 is more efficient and more responsive in their regulation of salt stress leading to higher tolerance. These are in line with previous studies comparing IR29 and FL478 [9, 14, 42, 43], IR29 and Pokkali [8, 38, 42], or other cultivars [44] associated with salt stress defense and related DAPs. The universal stress protein (Usp) is a multi-stress responsive protein conserved and provides stress resistance to relatively diverse organisms [45, 46]. The aldehyde dehydrogenase is active in detoxification, antioxidant and regulatory functions [47], but it catalyzes the dehydrogenation of D-glyceraldehyde 3-phosphate into 3-phospho-D-glycerate and NADH in rice. The aldehyde dehydrogenases are also involved in the oxidation of aldehydes to carboxylic acids in rice [48]. The detected glutaredoxin domain-containing protein detected in both IR29 and FL478 is involved in reducing GSH-thiol disulfides in rice associated with ion binding and transport. They are involved in the oxidative response in many plants directly reducing peroxides, dehydroascorbate, peroxidoxins and protecting thiol groups [49, 50]. The peptidyl-prolyl cis-trans isomerase detected in the present study can accelerate the folding of proteins. Two variants were detected in FL478 and the one located in the chloroplast is also associated with lateral root morphogenesis, photosystem II assembly and photosystem II stabilization. In higher plants, they assist in protein folding and those found in chloroplasts, mitochondria, and cytosol are light-controlled and are stimulated by heat, cold and high salt concentration [51] while they participate in extracellular matrix development on the cell surfaces [52]. The uncharacterized protein (A2X9N1) is related to ATP synthesis and ion transport. These proteins are potentially critical in IR29 and FL478 responding to severe salt stress conditions.

The common downregulated DAPs in both IR29 and FL478 are associated with diverse biological processes including photosynthesis (NADPH-protochlorophyllide oxidoreductase (OsI_17934), Clu domain-containing protein), transcription-related and chromosomal stability (histone H4), carbohydrate metabolism (sucrose synthase (OsI_11950), UDP-glucose 6-dehydrogenase (OsI_13603)), molecular chaperones (CCT-eta (OsI_24243) and metabolism (adenylosuccinate synthetase, chloroplastic (PURA), UDP-glucose 6-dehydrogenase (OsI_13603). In previous studies, there were a few detected common downregulated transcripts between IR29 and FL478 [14] and IR29 and Pokkali [8]. In this study, we have detected more commonly downregulated proteomes in IR29 and FL478 potentially due to their common response to severe salt stress conditions. In addition, our result showing the downregulation of many DAPs in both IR29 and FL478 potentially indicates harmful and possibly detrimental effects of severe salt stress on both genotypes.

Aside from the common significant DAPs between IR29 and FL478, genotype-specific significantly regulated DAPs also provide information on the proteomic responses of rice and the potential mechanisms of salt stress through proteomes. Most of the significantly upregulated DAPs in FL478 can be broadly categorized into four clusters particularly those related to carbohydrate and energy metabolism, photosynthesis, stress and defense, and metabolism. In IR29, protein synthesis proteins are abundant followed by stress and defense-related proteins, metabolism of carbohydrates and photosynthesis. In addition, most of the downregulated DAPs are also associated with the synthesis of protein, transcription, metabolism, metabolism of carbohydrates and photosynthesis. It can be noted that the biological processes with which most of the upregulated DAPs are associated are also the same as those that were downregulated particularly protein synthesis, carbohydrate metabolism and photosynthesis. This indicates that one of the responses to salt tolerance in IR29 seems to balance the processes greatly depreciated by salt stress by upregulating similar functional proteins that were downregulated by salt stress.

Aside from the stress and defense-related proteins discussed above commonly upregulated in both IR29 and FL478, there is a greater representation of FL478 genotype-specific proteomes associated with photosynthesis, carbohydrate metabolism and stress and defense. Photosynthesis is severely influenced by salt stress further developing to critically detrimental effects in plants [35]. Photosynthesis is linearly affected by increasing sodium concentration in the leaves particularly in older plants which concurrently affects stomatal aperture and gas exchange as well as carbon dioxide fixation [33, 53]. The osmotic effects of salt in the rhizosphere can also severely impact photosynthesis in plants [1, 54]. Looking at the specific proteins in FL478, there are more DAPs upregulated compared to a few DAPs in IR29 related to photosynthesis. These overrepresented photosynthesis-related DAPs are associated with thylakoid and chloroplast integrity and photosystem II assembly and stabilization. Salt-tolerant rice cultivars are known to more effectively manage photosynthesis-related aspects compared to their salt-sensitive counterparts [8] probably as a result of their higher chlorophyll pigment contents [14] leading to photosynthesis recovery under prolonged stress in contrast to the sensitive rice genotype [14]. These studies implicate that there is less severe stress in FL478 as observed in Pokkali [8].

### Inoculation with *Methylobacterium oryzae* CBMB20 Restored the Abundances and Potential Functions of Significantly Downregulated Proteomes under Severe Salt Stress Conditions

The application of *M. oryzae* CBMB20 profoundly changed the proteomes of IR29 and FL478 under an ideal growing environment [21]. One of the most prominent changes observed after inoculating IR29 and FL479 followed by salt stress imposition is the ‘restoration’ of function of proteins. We have observed common and genotype-specific DAPs that were upregulated in salt-stressed rice plants applied with *M. oryzae* CBMB20 while their counterparts were severely downregulated in the salt-stressed, non-inoculated rice plants.

Both IR29 and FL478 showed three major DAPs with significant upregulation when they were applied with *M. oryzae* CBMB20 and then subsequently exposed to salt stress. Like our results, sucrose synthase and peroxidase enzymes were downregulated under salt stress conditions in IR29 and Pokkali [38] and IR29 [8]. Sucrose synthase is also downregulated due to salt-induced stress [55-57]. The peroxidase enzyme has unpredictable regulation in different plants under stress as it can be up- or downregulated. The third DAP, NADPH-protochlorophyllide oxidoreductase (OsI_17934) was observed to be downregulated in *Arabidopsis thaliana* leaf microsomal membrane due to salt-induced stress [55]. However, inoculation with *M. oryzae* CBMB20 restored the functions of these proteins as their abundance and fold change increased in both IR29 and FL478.

The DAP NADPH-protochlorophyllide oxidoreductase (OsI_17934) is involved in the phototransformation of protochlorophyllide to chlorophyllide, an essential process in the transformation of chloroplast from etioplasts [58]. This enzyme was also upregulated in non-stressed, *M. oryzae* CBMB20 inoculated IR29 [21]. Our current result showed very high upregulation of this DAP due to *M. oryzae* CBMB20 in salt-stressed rice plants. Plant pigments (chlorophyll a, b, and carotenoids) were significantly increased under normal and under salt stress in a japonica cultivar inoculated with *M. oryzae* CBMB20 [22]. Photosynthetic rate and PSII efficiency in IR29 and FL478 also improved due to *M. oryzae* CBMB20 [36]. These improvements in the photosynthetic traits under salt stress when plants were applied with *M. oryzae* CBMB20 could be due to the upregulation and restoration of DAPs associated with the chloroplast or photosynthesis as in the case of NADPH-protochlorophyllide oxidoreductase.

Sucrose synthase (OsI_11950) cleaves sucrose to UDP-glucose and fructose. The end products of sucrose breakdown by sucrose synthase become available to diverse metabolic pathways including generation of energy, production of primary metabolites and carbohydrate biosynthesis [59]. In rice, it is involved in some housekeeping roles, cellulose synthesis, carbon allocation, and stress response [60]. The upregulation of two variants of sucrose synthase in both IR29 and FL478, Sucrose synthase (OsI_11950) and Sucrose synthase (OsI_22003) indicates a potential improvement or restoration of functions attributed mainly to carbohydrate metabolism and energy production. In a previous study in salt-stressed *Oryza sativa* ssp. *japonica* inoculated with *M. oryzae* CBMB20, the majority of DAPs belong to the carbohydrate metabolic process [22]. A similar observation can also be seen in this study.

The peroxidase upregulated in IR29 is mainly involved in the catabolism of hydrogen peroxide and oxidative stress response while the two peroxide variants observed in FL478 are also involved in lignin degradation and synthesis, catabolism of auxin, and response to stresses including invasion of pathogens, oxidative damage and physical damage. Upregulation of peroxidase in Pokkali was mainly observed during the stress compensation period where upregulation of defense against reactive oxygen species predominates [38]. In addition, the peroxidase enzyme observed in FL478 potentially contributes to lignin biosynthesis which could promote additional cell wall rigidity, similar to the observation in Pokkali [8]. The observed *M. oryzae* CBMB20-induced upregulation of antioxidant activities protecting plants against salt stress was also seen in previous studies [61]. Supporting the functions of the peroxidase enzyme, the genotype-specific enzyme xyloglucan endotransglucosylase/hydrolase (OsI_28350) upregulated in the *M. oryzae* CBMB20 inoculated FL478 also contributes to cell wall reconstruction indicating that inoculation with *M. oryzae* CBMB20 under salt stress enhances cell wall reconstruction adding another layer of defense response to salt-induced stress. Pokkali can sustain better plasticity of the cell wall under salt stress compared to IR29 [8]. However, it seems that inoculation with *M. oryzae* CBMB20 has enhanced this vital response in FL478 observed in the current study.

The other genotype-specific DAPs whose abundances were significantly upregulated potentially restoring their activities are associated with different functions. In IR29, two DAPs are associated with protein synthesis: the ribosomal component 40S protein and the eukaryotic translation initiation factor, and another DAP is associated with purine nucleotide biosynthesis (adenylosuccinate synthetase). On the other hand, the genotype-specific DAPs with restored function in *M. oryzae* CBMB20 inoculated FL478 are involved with salt stress tolerance (fasciclin-like arabinogalactan protein), protein synthesis (NAC-A/B domain-containing protein), and one-carbon metabolism (methylenetetrahydrofolate reductase). The restoration of abundances of downregulated proteins due to the application of *M. oryzae* CBMB20 together with the other genotype-specific significantly upregulated DAPs indicates the essential impact of inoculation in rice similarly observed even under non-stress conditions [21] and in *Oryza sativa* ssp. *japonica* under severe salt stress [22].

### Mechanisms of Action Leading to the Dynamic Proteomic Responses of the *M. oryzae* CBMB20 Inoculated Rice Plants under Severe Salt Stress

**Fig. 7:**
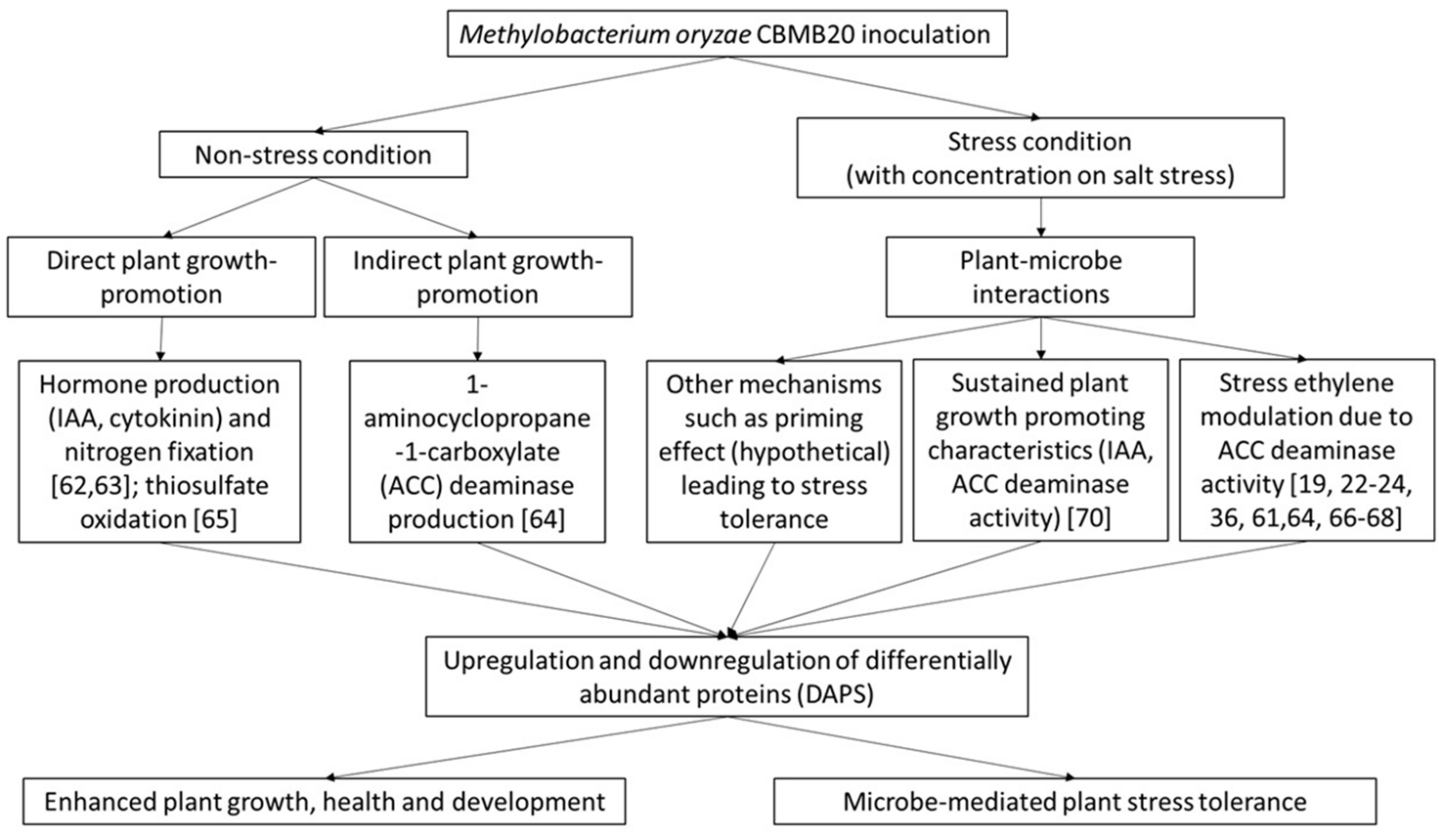
Hypothetical mechanisms of plant growth-promotion and microbe-mediated stress tolerance by *Methylobacterium oryzae* CBMB20.

*M. oryzae* CBMB20 is a multifunctional PGPB with growth-promoting abilities including N_2_ fixation, phytohormone production (IAA, cytokinin) [62, 63], 1-aminocyclopropane-1-carboxylate (ACC) deaminase production [64], and thiosulfate oxidation [65]. Under salt stress conditions, there are additional mechanisms of action by this multifaceted PGPB. Figure 7 shows the summary of the mechanisms of action of plant growth-promotion and microbe-mediated stress tolerance conferred by *M. oryzae* CBMB20 on inoculated plants as supported by previous investigations.

*M. oryzae* CBMB20 can enhance the health of stressed plants including pathogen attack [66-68], heavy metal stress [19], and salt stress [22-24, 36, 61]. This feat by *M. oryzae* CBMB20 is mainly due to its ACC deaminase activity under stress conditions. *M. oryzae* CBMB20 is capable of regulating stress ethylene by sequestering and hydrolyzing the ethylene precursor, ACC, to α-ketobutyrate and ammonia through the action of its ACC deaminase [64]. In our current investigation, the restoration of abundances of numerous downregulated DAPs under severe salt stress in both IR29 and FL478 after the application of *M. oryzae* CBMB20 is potentially due to the lowering of stress via ethylene reduction. This subsequently allowed IR29 and FL478 to recover and upregulate their DAPs initially downregulated due to salt stress. Aside from the action of ACC deaminase, it is also still possible that *M. oryzae* CBMB20 can maintain plant growth promotion through the direct mechanisms mentioned above even under stress conditions.

Another potential mechanism as recently supported by a study [24] is the involvement of ethylene-induced pathogenesis related signaling, where priming may also be involved. In priming, the sensitization of the whole plant for stronger defense responses, mainly characterized by faster, more robust and more efficient activation of cellular defenses is well established in PGPB inoculated plants subsequently challenged by pathogens or attacked by herbivores [69]. The enhanced protection of *M. oryzae* CBMB20 on pathogen-challenged plants was already observed particularly against *R. solanacearum* [67], *Xanthomonas campestris* [68], and *P. syringae* pv. tomato [66], although all these studies have mainly attributed this to ACC deaminase and the regulation of stress ethylene. In a recent study, stress and defense responses were activated and modulated by the application of *M. oryzae* CBMB20 under normal conditions in both IR29 and FL478 when *M. oryzae* CBMB20 endophytically colonized the rice plants [21]. We are proposing that stress and defense responses have been primed due to the application of the *M. oryzae* CBMB20 leading to a more effective response when the rice cultivars were subsequently exposed to severe salt stress resulting to the observed dynamic proteomic responses observed in this study.

## Conclusion

Salt stress has a profound influence on the proteomes of both the salt-sensitive IR29 and the moderately salt-tolerant FL478 rice genotypes. Under severe salt stress conditions, the proteomes of rice in terms of abundance and identity are mainly influenced by salt stress, rice genotypes, and the application of *M. oryzae* CBMB20. Interestingly, the endophytic PGPB *M. oryzae* CBMB20 mediated changes in the salt-stressed salt-sensitive IR29 resulting in similar proteomes to that of the salt-tolerant FL478. Severe salt stress resulted in the downregulation of many protein abundances in IR29 and FL478. There are common upregulated and downregulated DAPs in both IR29 and FL478 due to salt stress indicating similar mechanisms of salt stress responses and similar biological and molecular processes negatively influenced by salt stress conditions. Nevertheless, the moderately salt-tolerant FL478 upregulated more proteins with higher fold change values indicating more efficient responses to salt stress. The application of the two rice genotypes with the multifaceted plant growth-promoting *M. oryzae* CBMB20 mediated proteomic changes under salt stress conditions. The presence of common DAPs between the *M. oryzae* CBMB20 inoculated IR29 and FL478 indicates common mechanisms of microbe-mediated salt stress tolerance. A key feature of the *M. oryzae* CBMB20-mediated salt stress responses is the restoration of the abundance of many downregulated DAPs in both genotypes aside from the other significantly upregulated DAPs indicating its critical importance in imparting microbe-mediated salt stress tolerance.

## Supplemental Materials

Supplementary data for this paper are available on-line only at http://jmb.or.kr.



## Figures and Tables

**Table 1:** Common DAPs in IR29 and FL478 under 200 mM salt stress.

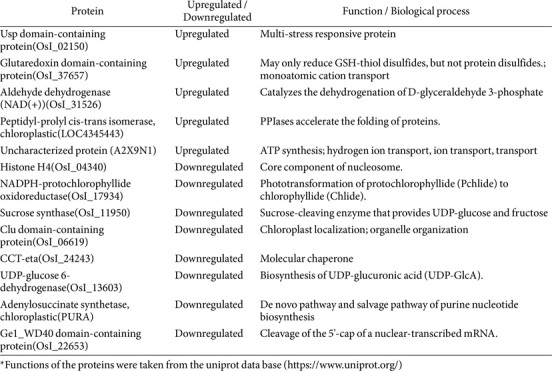

**Table 2:** Common and genotype-specific DAPs that were downregulated under salt stress and were upregulated due to CBMB20 inoculation.